# Three Cases of Hemiconvulsion-Hemiplegia-Epilepsy Syndrome With Focal Cortical Dysplasia Type IIId

**DOI:** 10.3389/fneur.2019.01233

**Published:** 2019-11-20

**Authors:** Shinji Itamura, Tohru Okanishi, Yoshifumi Arai, Mitsuyo Nishimura, Shimpei Baba, Naoki Ichikawa, Yoshimichi Hirayama, Naoko Ishihara, Takuya Hiraide, Hidetoshi Ishigaki, Tokiko Fukuda, Yoshiro Otsuki, Hideo Enoki, Ayataka Fujimoto

**Affiliations:** ^1^Department of Child Neurology, Seirei-Hamamatsu General Hospital, Shizuoka, Japan; ^2^Department of Pathology, Seirei-Hamamatsu General Hospital, Shizuoka, Japan; ^3^Department of Clinical Laboratory, Seirei-Hamamatsu General Hospital, Shizuoka, Japan; ^4^Epilepsy Center, Seirei-Hamamatsu General Hospital, Shizuoka, Japan; ^5^Department of Pediatrics, Naha City Hospital, Okinawa, Japan; ^6^Department of Pediatrics, Fujita Health University School of Medicine, Toyoake, Japan; ^7^Department of Pediatrics, Hamamatsu University School of Medicine, Hamamatsu, Japan

**Keywords:** hemiconvulsion-hemiplegia-epilepsy syndrome, hemispherotomy, focal cortical dysplasia, cortical malformation, intractable epilepsy, epilepsy surgery, pathology, histopathology

## Abstract

Hemiconvulsion-hemiplegia-epilepsy syndrome (HHES) is a subset of acute encephalopathy characterized by infantile-onset with acute hemiconvulsive febrile status and subsequent unilateral cerebral atrophy and hemiparesis. In the chronic phase, patients with HHES develop epilepsy, typically displayed as intractable focal seizures. The patients are often intractable with antiepileptic drugs and need surgical treatment. Although viral encephalitis and genetic abnormalities are presumed to be the underlying etiology, the pathogenesis remains mostly unknown. We describe three cases of successful functional hemispherotomy for intractable epilepsy in HHES. Patients developed acute asymmetrical convulsive status following viral infections during the ages of 17–30 months. Their seizures were intractable with antiepileptic drugs and required hemispherotomy. On the basis of the pathological findings, all cases were diagnosed as focal cortical dysplasia (FCD) type IIId. The epileptogenic mild cortical malformations may be the cause of HHES.

## Introduction

Hemiconvulsion-hemiplegia-epilepsy syndrome (HHES) is a rare condition first described by Gastaut et al. in 1960 (1) that starts with virus infection-associated acute encephalopathy in the unilateral hemisphere. This condition is characterized by prolonged febrile status of hemiconvulsion (asymmetrical convulsion) in the acute phase, and development of hemiparesis/hemiplegia and intractable epilepsy in the chronic phase. Hemispheric cytotoxic edema occurs in the acute phase and is presented as reduced diffusivity in brain magnetic resonance imaging (MRI), including diffusion-weighted imaging (DWI). The affected hemisphere evolves into atrophy in the chronic phase. Only a few pathologic investigations have been reported; therefore, the pathogenesis of HHES has not yet been determined (2–5).

Focal cortical dysplasia (FCD) is an epileptogenic cortical tissue frequently seen in the resected cortices of patients who have undergone epilepsy surgery. In the 2011 International League Against Epilepsy (ILAE) classification, FCD was classified into type I: dyslamination and disrupted organization of tissue architecture with morphologically normal neurons and glial cells; type II: presence of dysplastic, megalocytic neurons mixed with normal neurons; and type III: adjacent to another principal lesion. Type IIId is diagnosed in association with epileptogenic lesions acquired early in life (6).

We herein report three cases of HHES with FCD type IIId in the affected cortices.

## Case Presentation

We summarized the clinical information of the three cases in Table 1.

**Table 1:** Clinical information and pathological findings in the 3 cases.

	**Case 1**	**Case 2**	**Case 3**
Age at onset of acute encephalopathy	18 mo	28 mo	17 mo
Side of affected hemisphere	Right	Right	Left
Type of convulsive status at onset	Left-sided clonic seizures	Left-sided clonic seizures	Right-sided clonic seizures
Causative virus	Enterovirus	Influenza type A	Human herpesvirus 6
DWI findings in the acute phase (d of onset)	Subcortical high intensities diffusely in the right hemisphere (3d)	Cortical and subcortical high intensities diffusely in the right hemisphere (5d)	Cortical and subcortical high intensities diffusely in the left TPO (3d)
Treatment for acute encephalopathy	mPSL, IV	mPSL, IV, mannitol, PB, MDZ	mPSL, MDZ
Antiepileptic drugs before surgery	CLB, LTG, TPM, CBZ, LEV	PB, VPA, CLB, PER, LTG, LEV	VPA, CLB, LTG, PER, TPM, LEV
MRI findings in the chronic phase	Diffuse atrophy and hypoperfusion in the right hemisphere	Diffuse atrophy and hypoperfusion in the right hemisphere	Diffuse atrophy in the left hemisphere
Development quotient before surgery (age)	46 (39 mo)	31 (56 mo)	34 (56 mo)
Motor disability before surgery	Left hemiparesis	Left hemiparesis	No paresis
Seizure types before surgery (frequency)	1: Left side dominant-tonic spasms (10/d); 2: Myoclonic seizures (>30/d)	Left side dominant-tonic spasms (2–5/d)	1: Focal impaired awareness seizures with foci in left F, O; 2: Asymmetrical tonic seizure secondary to seizure 1
Ictal EEG findings	1: Diffuse spike burst followed by polyphasic slow waves; 2: Diffuse polyspikes and waves	Diffuse attenuation in the right hemisphere	1: Frequent spikes and theta burst in left F or O; 2: Fast wave -> spike burst in left hemisphere
Age at epilepsy surgery	41 mo	59 mo	60 mo
Type of epilepsy surgery	Right hemispherotomy	Right hemispherotomy	Left subtotal hemispherotomy
Seizure outcomes of the surgery (follow-up period)	Seizure free (10 mo)	Seizure free (11 mo)	Seizure free (10 mo)
Pathology			
Neocortex	Excessive neuronal cells and glial cells in white matter (FCD type IIId) in temporal lobe	Excessive neuronal cells and glial cells in white matter (FCD type IIId) in temporal lobe	Excessive neuronal cells and glial cells in white matter (FCD type IIId), immature-like in temporal lobe
Amygdala	No change	No change	Immature-like neurons
Hippocampus	No change	No change	Gliosis

*y, year(s); mo, month(s); d, day(s); mPSL, methylprednisolone; IV, immunoglobline; MDZ, midazolam; CLB, clobazam; LTG, lamotrigine; TPM, topiramate; CBZ, carbamazepine; LEV, levetiracetum; PB, phenobarbital; VPA, valproic acid; PER, perampanel; F, frontal lobe; T, temporal lobe; O, occipital lobe; FCD, focal cortical dysplasia*.

### Case 1

An 18-month-old boy with a healthy clinical history presented with left-sided status of clonic convulsion lasting over 30 min following fever. Enterovirus was isolated from his pharyngeal secretion on the day of admission. Three days following onset, brain DWI showed reduced diffusivity on subcortical white matter in the right hemisphere. He was diagnosed with viral infection associated-acute encephalopathy and treated with methylprednisolone pulse therapy, high dose immunoglobulin and continuous intravenous midazolam. After the acute phase of the encephalopathy, he presented with left hemiparesis. Left side dominant-tonic spasms recurred 3 months following onset. Despite treatment with clobazam, lamotrigine, topiramate, carbamazepine, and levetiracetam, the seizures continued to occur daily.

He was referred to our epilepsy center at the age of 39 months. He had left hemiparesis and could only speak a few words. His developmental quotient (DQ) was 46 on the Kinder Infant Development Scale. Long-term video-electroencephalogram (EEG) monitoring revealed that the left side dominant-tonic spasms (10/day) coincided with ictal changes of diffuse spike bursts followed by biphasic or triphasic diffuse slow waves, and that the myoclonic seizures (>30/day) coincided with diffuse polyspikes and waves. Brain MRI revealed right cerebral atrophy and hypoperfusion with arterial spin labeling (ASL). At the age of 41 months, he underwent right hemispherotomy. He was free from any seizures up to the last follow-up at 10 months after the surgery. The left hemiparesis transiently worsened after the surgery and then recovered to the presurgical level. His attention and cognitive function improved.

The histopathological examination with hematoxylin-eosin staining revealed an excessive number of neurons and glial cells in the white matter with intact cortical architecture and absence of aberrant cells, corresponding to FCD type IIId, on the right tip of the temporal lobe (Supplementary Figures 1A,B, 2A). No changes were observed in the right amygdala and hippocampus.

### Case 2

**Figure 1:**
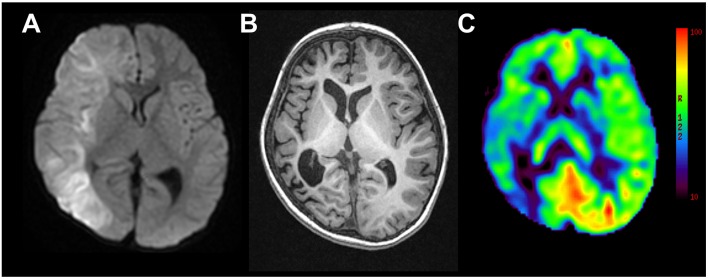
Representative magnetic resonance images from case 2. **(A)** Diffusion weighted imaging at day 5 following onset of left-sided convulsion revealed high signals in subcortical areas in the right hemisphere. **(B)** T1-weighted imaging 2 years following onset revealed right hemispheric atrophy. **(C)** Arterial spin labeling at the same time revealed hypoperfusion in the right hemisphere.

A 28-month-old boy with a healthy clinical history presented with left-sided status of clonic convulsions lasting >30 min with fever. His pharynx was positive for the antigen of influenza virus type A. Brain DWI showed reduced diffusivity in the cortex and subcortical white matter in the right hemisphere, at 5 days after the occurrence (Figure 1A). He was treated with methylprednisolone pulse therapy and high dose immunoglobulin, mannitol, phenobarbital, and continuous intravenous midazolam. After encephalopathy onset, he presented with left hemiparesis, and suffered from left side dominant- or symmetrical tonic spasms. Although he was treated with phenobarbital, valproic acid, clobazam, perampanel, lamotrigine, and levetiracetam, seizures continued to occur daily.

He was referred to our epilepsy center at the age of 56 months. DQ was 31 on the Kyoto Scale of Psychological Development and he presented with left hemiparesis. Long-term video-EEG revealed left side dominant-tonic spasms and ictal attenuation in the right hemisphere with a frequency of 2–5/day. Brain MRI revealed right hemispheric atrophy and right hemispheric hypoperfusion with ASL (Figures 1B,C). At the age of 59 months, he underwent right hemispherotomy. He was free from any seizures up to 11 months after the surgery. The left hemiparesis worsened transiently after the surgery and then recovered to the presurgical level.

The histopathological examination with hematoxylin-eosin staining revealed an excessive number of neurons and glia cells in the white matter with intact cortical architecture and absence of aberrant cells, corresponding to FCD type IIId, in the cerebral tissues of the tip of the right temporal lobe (Supplementary Figures 1C,D) and adjacent to the right hippocampus. No changes were observed in the right amygdala and hippocampus.

### Case 3

A 17-month-old boy with a healthy clinical history presented with left-sided status of clonic convulsion lasting 60 min. Human herpesvirus 6 was isolated from his pharynx. He was treated with methylprednisolone pulse therapy and continuous intravenous midazolam. Three days following onset, cortical-subcortical restricted diffusivity was observed on the left temporo-parieto-occipital lobes in brain DWI. Following the acute phase, he continuously developed focal seizures accompanied by impaired consciousness and cyanosis. Despite treatment with valproic acid, clobazam, lamotrigine, perampanel, topiramate, and levetiracetam, daily focal seizures accompanied by mouthing, grimacing or expressions of fear continued to occur. Two months following onset, brain MRI revealed left hemispheric atrophy.

He was referred to our epilepsy center at the age of 56 months. He was not paralyzed but was barely able to speak. DQ was 34 on the Kyoto Scale of Psychological Development. Long-term video-EEG monitoring revealed that the focal impaired awareness seizures coincided with ictal EEG activities from the left frontal and occipital areas, which sometimes evolved into asymmetrical tonic seizures (extensions of left extremities and flexion of right extremities). Left hemispheric atrophy was observed in brain MRI. At the age of 60 months, he underwent left subtotal hemispherotomy (hemispherotomy sparing the pyramidal tract). He was free from seizures up to the last follow-up 10 months after surgery.

**Figure 2:**
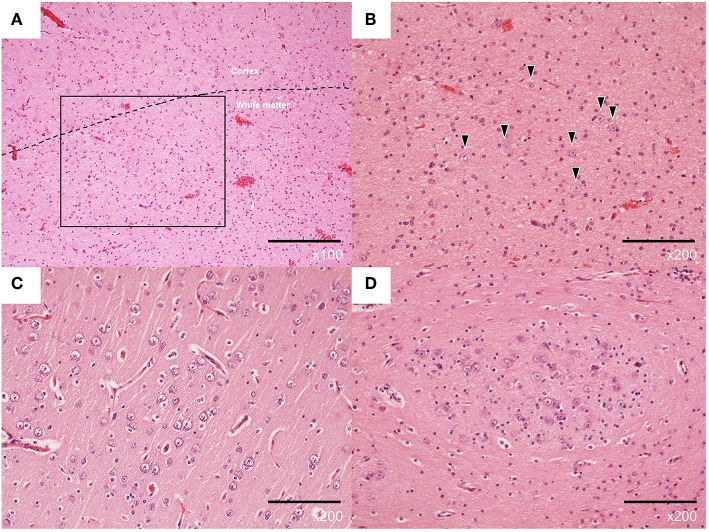
Representative pathological findings with hematoxylin-eosin staining of the left lateral temporal lobe **(A–C)** and left amygdala **(D)** from case 3. **(A)** Normal architecture of cortical and subcortical white matter (×100). **(B)** Excessive number of neurons in the white matter (×200, arrowheads). **(C)** Neurons with increased nuclear/cytoplasmic ratio in the cortex (×200). **(D)** Clustered immature neurons (×200).

The histopathological examination with hematoxylin-eosin staining revealed immature-like neurons with large nuclei in the cerebral cortex of the tip of the left temporal lobe (Supplementary Figure 2B). An excessive number of neurons and glial cells was observed in the white matter of the same region, corresponding to FCD type IIId (Figures 2A–C). Clustering of immature-like neurons was observed in the left amygdala (Figure 2D) and gliosis was observed in the right hippocampus.

### Procedures of Hemispherotomy

We performed the hemispherotomies using a standard peri-insula approach, which disconnected the association and commissural fibers in one hemisphere and interrupted the radiation fibers. Medial temporal structures, including the hippocampus and amygdala, were resected, along with the circular sulcus, except in case 3. In case 3, we performed posterior quadrant disconnection, with frontal lobe disconnection preserving the pyramidal tract to secure fine finger motor movement, such as pincer movement.

## Discussion

We reported 3 cases of HHES following virus infection associated-acute encephalopathy. Patients suffered from intractable seizures in chronic periods, which were completely resolved by functional hemispherotomy. The histopathological examinations revealed FCD type IIId in the cortices of all cases. One case presented additional aberrant neurons in the neocortex and amygdala.

Some etiologies have been described as the cause of HHES. Viral infections, including human herpesvirus 7, varicella zoster virus, and other viruses, have been reported as the trigger of HHES (7, 8). Genetic background has also been reported. Mutations in *SCN1A* and *CACNA1A*, the causative genes of Dravet syndrome or febrile seizures and familial paralytic migraine headache, respectively, have been reported in HHES patients. The excessive epileptic excitability of the cortex conferred by these mutations may also contribute to the development of HHES (9, 10). However, why the excitation and damage occur only in unilateral hemisphere remains unexplained.

There are only a few reports of pathological investigations regarding HHES. Mori reported neuropathological studies of 17 HHES patients who underwent hemispherectomy (2, 3). One patient had polymicrogyria in the left sylvian fissure and another presented non-specific findings of diffuse cortical scarring in the laminae and demyelination of the white substance. Auvin et al. (4) reported post-mortem specimens without any cell death in the cerebrum as a result of prolonged seizures. The last report was the biopsy case of the right temporal cortex in a patient with HHES in need of decompressive craniectomy. The histopathological examination revealed reduced granular cells in diffuse spongiosis and edema without cell necrosis in the cortical tissue (5). Thus, previous reports indicate no specific pathological findings, other than the case of polymicrogyria.

On the other hand, all our cases showed FCD type IIId. FCD type IIId is observed in the cortical tissues in resective surgical cases of intractable epilepsy and Rasmussen encephalitis, and the aberrant structure has the potential to induce seizures (11, 12). Our cases indicate that the fever associated with the viral infections triggered the seizures in the hemisphere with FCD type IIId and induced epileptic excitotoxicity. Epileptic excitotoxicity during infant to early childhood can result in cytotoxic edema as observed by DWI and secondary atrophic changes (13). The mild cortical malformation and additional episodes of neuroexcitation may be one of the main causes of HHES. The lack of reporting of these findings in the previous study might be due to the recent establishment of the classification and lack of recognition.

Hemispheric cortical malformation with epileptic excitability has been reported to be caused by somatic mutations related to cortical dysplasia in the affected hemisphere (14). Our cases suggest that somatic mutations related to FCD might increase the susceptibility of developing virus infection associated-acute encephalopathy in the unilateral hemisphere that result in hemispheric involvement. More genetic investigations and pathological cases are necessary to clarify the correlation between mild cortical malformation and emergence of HHES.

In conclusion, we reported 3 cases of HHES likely caused by FCD type IIId in the affected hemisphere.

## Ethics Statement

The studies involving human participants were reviewed and approved by Ethical board of Seirei Hamamatsu General Hospital. Written informed consent to participate in this study was provided by the participants' legal guardian/next of kin.

## Author Contributions

SI, TO, HE, and AF contributed to conceptualizing, drafting, and revising the study. YA and YO contributed to analyzing and interpreting the pathological examinations. MN, SI, TO, and SB contributed to the interpretation of EEG data. SI, TO, SB, NIc, YH, NIs, TH, HI, and TF contributed to acquiring and the interpreting the clinical information and MRI data.

### Conflict of Interest

The authors declare that the research was conducted in the absence of any commercial or financial relationships that could be construed as a potential conflict of interest.

## Supplementary Material

Supplementary Figure 1 Pathological findings with hematoxylin-eosin staining of affected lateral temporal lobes in case 1 [**A** (×100), **B** (×400)] and 2 [**C** (×100), **D** (×400)]. Normal architecture of cortex and subcortical white matter **(A,C)**. Neurons are ectopically observed in the white matter **(B,D)**.

Supplementary Figure 2 Location of the cortical tissues resected for pathology in case 1 **(A)** and 3 **(B)** (Circles).
