# Supplementary figures and images for: Three Cases of Hemiconvulsion-Hemiplegia-Epilepsy Syndrome With Focal Cortical Dysplasia Type IIId
Source: Front Neurol. 2019 Nov 20;10:1233. doi: 10.3389/fneur.2019.01233 (PMC6879674; doi:10.3389/fneur.2019.01233)

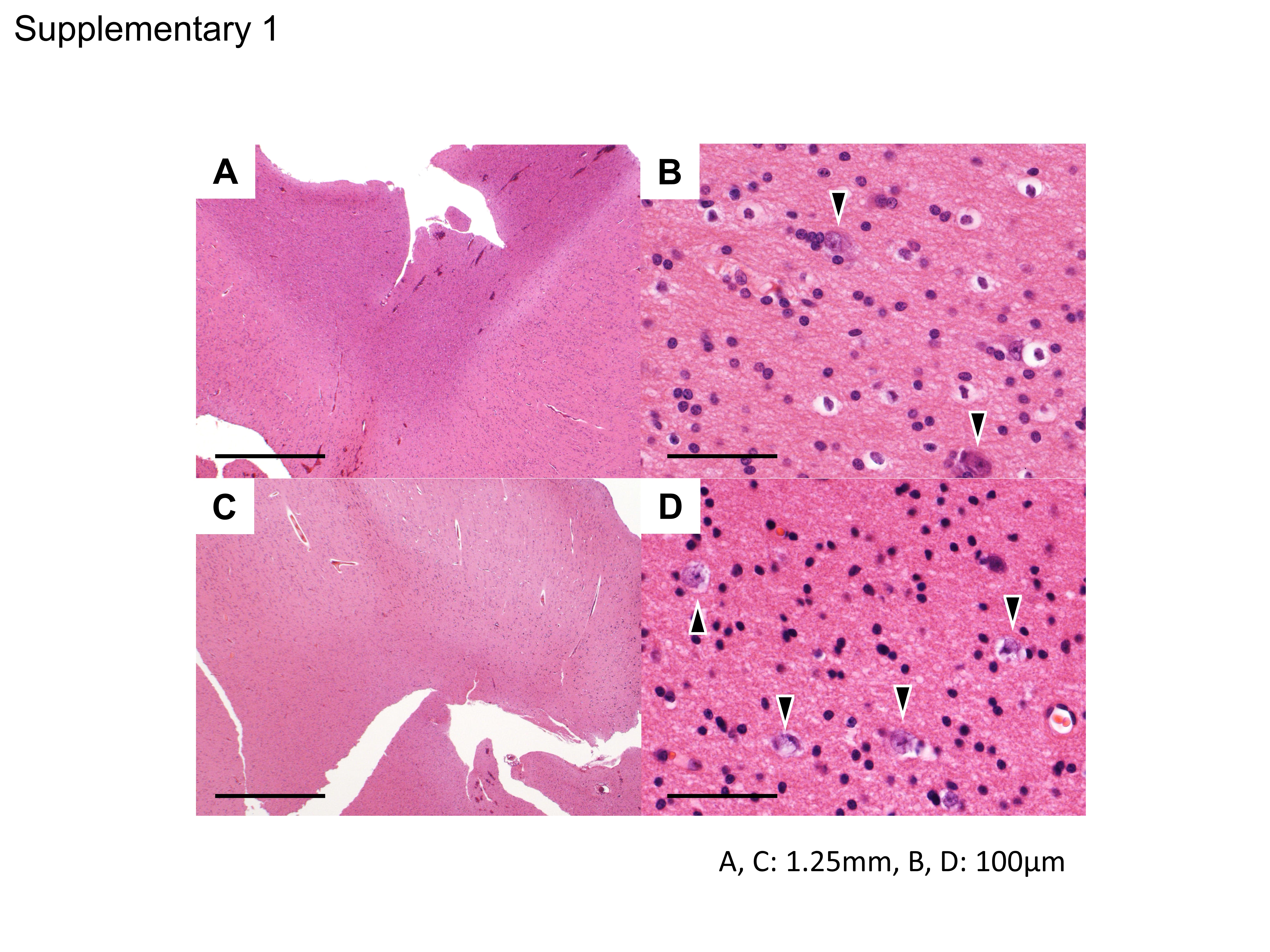

Supplement: Supplementary Figure 1 — Pathological findings with hematoxylin-eosin staining of affected lateral temporal lobes in case 1 [A (×100), B (×400)] and 2 [C (×100), D (×400)]. Normal architecture of cortex and subcortical white matter (A,C). Neurons are ectopically observed in the white matter (B,D). [file Image_1.JPEG]

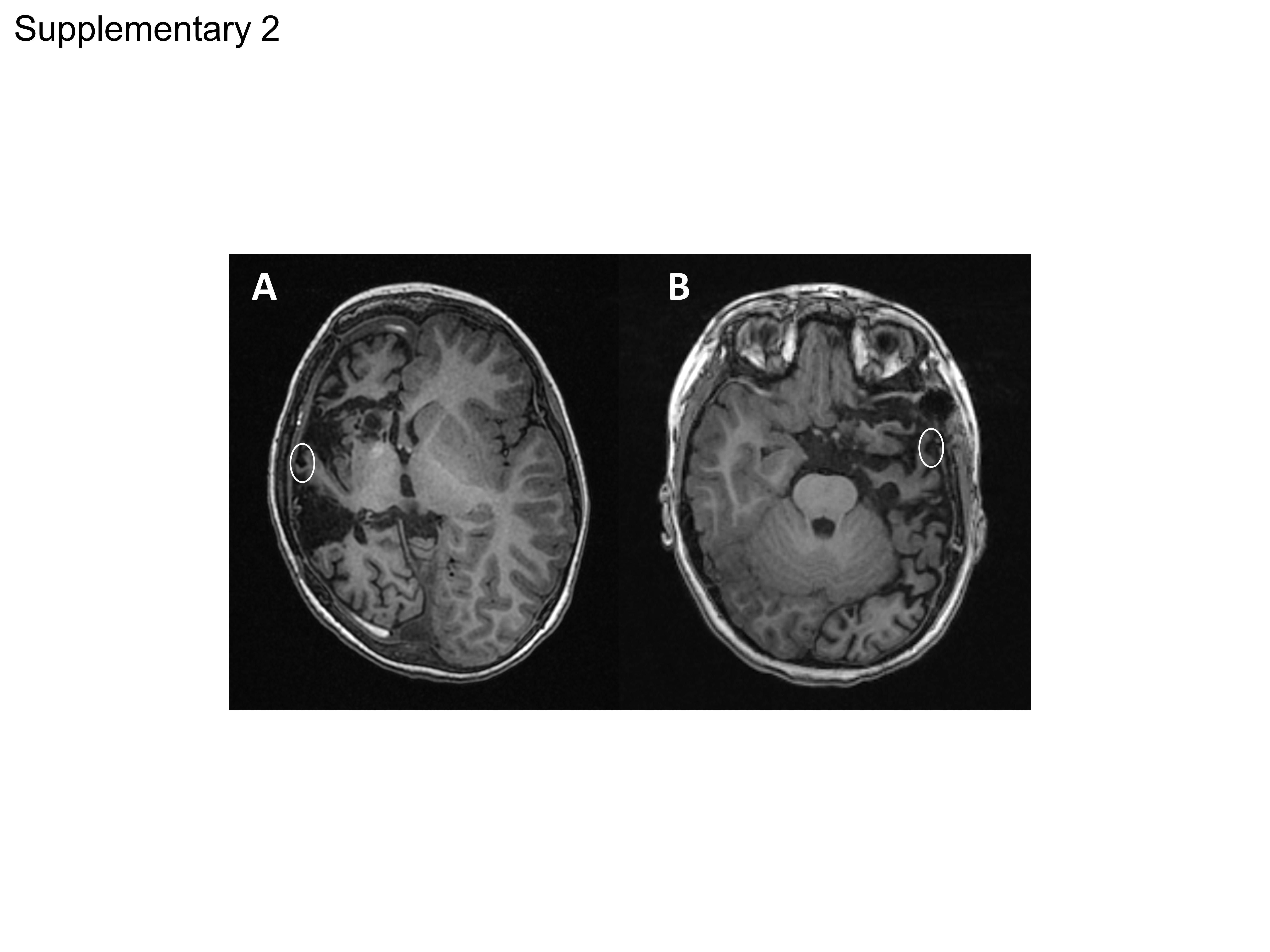

Supplement: Supplementary Figure 2 — Location of the cortical tissues resected for pathology in case 1 (A) and 3 (B) (Circles). [file Image_2.JPEG]
